# Editorial: Towards Real World Impacts: Design, Development, and Deployment of Social Robots in the Wild

**DOI:** 10.3389/frobt.2020.600830

**Published:** 2020-12-03

**Authors:** Chung Hyuk Park, Raquel Ros, Sonya S. Kwak, Chien-Ming Huang, Séverin Lemaignan

**Affiliations:** ^1^Department of Biomedical Engineering, School of Engineering and Applied Science, The George Washington University, Washington, DC, United States; ^2^Grup de Tecnologies Media, La Salle-Universitat Ramon Llull, Barcelona, Spain; ^3^Center for Intelligent and Interactive Robotics, Korea Institute of Science and Technology (KIST), Seoul, South Korea; ^4^Department of Computer Science, Johns Hopkins University, Baltimore, MD, United States; ^5^Bristol Robotics Laboratory, Bristol, United Kingdom

**Keywords:** social robots in the wild, HRI theories, HRI computational modeling, longitudinal HRI, large-group HRI, HRI in healthcare and special education, emotions and ethics in HRI

## Introduction

Social robots have great potential to provide social, behavioral, emotional, and cognitive support to people with diverse characteristics and needs. Although still in its infancy, the field of social robotics has explored various aspects of human-robot interaction (HRI), such as multimodal communication and personalized interaction, and their applications in different domains including education and patient care. However, to evaluate the acceptance and efficacy of social robots and to understand their broader impacts in the real world, it is necessary to deploy these robots in the “wild” field for an extended period of time. Such deployment typically involves collaboration with different disciplines such as medicine, social psychology, clinical therapy, industrial design, public health, marketing, and education.

Thus, this Research Topic focuses on social robotics research with novel algorithms and computational modeling that have been or are being evaluated with intended users/consumers, patients, or individuals with special needs. A special focus has been given to results that arose from multidisciplinary studies in which the roles and impacts of social robots are evaluated in “real-world” settings, especially in collaboration between engineering, industrial design, clinical science, medicine, social psychology, marketing, and education.

## Research Topic Formation

This Research Topic emerged from a discussion among young and active researchers in HRI—Dr. Park and Dr. Huang from the U.S.A., Dr. Ros from Spain, Dr. Kwak from South Korea, and Dr. Lemaignan from the United Kingdom—who have all realized the emergent and crucial needs to explore the topic further and get further input from the many researchers in HRI.

## Contents of the Research Topic

As anticipated, the submitted and accepted papers showed multi-disciplinary characteristics and combined themes from the proposed Research Topic outline. Out of the multiple mixed themes, however, we could identify the following four major important research themes and trends, where the social robots are taking active roles in the “wild” of the human-robot interaction frontiers.

### HRI Theories and Computational Modeling

We have seen continuous efforts in applying theories from psychology with more sound experimental settings in the real world. One recent example is by Agrigoroaie et al. who have applied the regulatory focus theory in human-robot communication, with which a Tiago robot approached people with two communication methodologies based on either promotion type or prevention type, and evaluated its effectiveness in correlation with the regulatory focus types of the individuals (*N* = 29).

We have also found increased efforts in designing computational models for HRI in the wild, especially in the clinical domains. Clabaugh et al. propose a math tutoring system for children with Autism Spectrum Disorder (ASD) (aged 4–7), where Reinforcement Learning is used to personalize instruction and robot feedback. Javed et al. apply machine learning algorithms to derive personalized models for acquiring social engagement measures for children with ASD (aged 4–12).

### Longitudinal and Large-Group HRI

For the past 10 years, longitudinal and larger-group studies in HRI have gained great interest from the community aiming at building systems that can engage and adapt to different users through time with the ambition of getting closer to a world where robots can truly be part of our daily lives. The educational setting is one of the typical areas where the application of social robots has been studied. A math tutoring system proposed by Clabaugh et al. has been evaluated with 17 children with ASD over month-long interventions at their homes. A peer-like social robot for language learning designed by Kory-Westlund and Breazeal has been developed, and the role of “rapport” has been investigated in their 2 months long study with 17 children. Besides education, social robots are used for elderly care; Van Maris et al. explored the longitudinal effects of older adults (*N* = 17) interacting with social robots.

HRI in public, especially interacting with a large group of people is another emerging Research Topic. Fraune et al. have studied the impact of group characteristics and norms in interacting with a robot in public settings and its influences on people's behavior changes.

### HRI in Healthcare and Special Education

ASD is one of the areas in special education where socially assistive robot (SAR) systems have shown potential benefits, typically supporting social development, though not limited to. An example is a work proposed by Clabaugh et al., where they propose a tutoring system for space-themed mathematics problems addressed to young children with ASD while embedding social contexts in the learning environment. Another work by Javed et al. exhibits a robotic playmate with socio-emotional interventions with personalized engagement monitoring.

Management of diabetes is another healthcare-related domain where in-the-wild long-term interventions are key. Neerincx et al. present the results of a 4-years European project, where they deploy the Socio-Cognitive Engineering methodology to design and integrate a SAR, tested for several months with large groups of children.

Though evaluating SAR systems with end-users is vital, it is equally important to consider the views of other stakeholders to guide the design of such systems. As such, Alcorn et al. present an analysis of educators' views on the use of SAR systems in ASD suggesting guidelines to the HRI community, not only regarding the design of robotic systems but also proposing areas of research that should be further considered.

### Emotions and Ethics

While socially assistive robots are uniquely characterized by their potential in participating in social-emotional interactions with people, these robots are currently not as emotionally capable as humans do. Indeed, emotive behaviors displayed by robots can be considered as emotional deception, possibly leading to broader ethical concerns. Van Maris et al. studied how deceptively emotive behaviors by a social robot might influence older adult's perceptions of the robot.

On the other hand, social robots' behaviors could possibly help mitigate human negative psychological states such as stress. Björling et al. explored how teens may interact with social robots in the school environment where teens might feel stressed. Little research on robotics and teens has been conducted to date: this article, relying on mixed-methods where the teens are in turn users, experimenters, and witnesses, offers a very novel glimpse into how socially assistive robots could support this population.

## Conclusions

With the development of robotic technologies, it is imperative to develop social robots that support people in their daily lives. The papers published in this issue collectively show the recent and advanced application of theories from various academic fields such as healthcare, education, social psychology on social robots and HRI. More efforts are found to be focusing on child/adolescents and older adults who are in need of or can benefit from the company of robots. We believe that these collective endeavors will help to further extract knowledge regarding the nature of the interaction between humans and robots, which we hope to be utilized in building successful social robots “in the wild,” expanding into wider areas of applications in diverse human domains (age, gender, culture, etc.). Specifically, topics presented by the papers including HRI Theories and Computational Modeling, Longitudinal and Larger-group HRI, HRI in Healthcare and Special Education, and Emotions and Ethics provide the readers with a vast array of applicable areas of social robots, focused on their effectiveness in various real-world settings. These studies will enlighten further research avenues regarding social robots that are closely connected to the users, which enrich their satisfaction with their lives by successfully fulfilling their needs. We believe that these collaborative efforts will contribute to the further development of the theory and knowledge regarding HRI, boosting the people's acceptance of social robots in their daily lives.

## Author Contributions

CP and C-MH conceived the idea for the Research Topic and recruited RR, SSK, and SL to form a team of guest editors for this Research Topic. Together, this team of guest editors have crafted the Research Topic abstract and the international call for papers. The editors have reviewed submitted papers within their expertise and accepted nine papers for this Research Topic. This editorial has been compiled through joint efforts of all five guest editors. All authors contributed to the article and approved the submitted version.

## Conflict of Interest

The authors declare that the research was conducted in the absence of any commercial or financial relationships that could be construed as a potential conflict of interest.

